# Mapping the breeding sites of *Anopheles gambiae* s. l. in areas of residual malaria transmission in central western Senegal

**DOI:** 10.1371/journal.pone.0236607

**Published:** 2020-12-11

**Authors:** Assane Ndiaye, El Hadji Amadou Niang, Aminata Niang Diène, Mohamed Abderemane Nourdine, Pape Cheikh Sarr, Lassana Konaté, Ousmane Faye, Oumar Gaye, Ousmane Sy

**Affiliations:** 1 Faculté des Lettres et Sciences Humaines, Département de Géographie, Université Cheikh Anta Diop de Dakar, Dakar, Sénégal; 2 Faculté des Sciences et Techniques, Laboratoire d’Ecologie Vectorielle et Parasitaire, Université Cheikh Anta Diop, Dakar, Sénégal; 3 Faculté de Médecine, Laboratoire de Parasitologie médicale, Pharmacie et d’Odonto-stomatologie, Université Cheikh Anta Diop, Dakar, Sénégal; Instituto Rene Rachou, BRAZIL

## Abstract

Despite the deployment of several effective control interventions in central-western Senegal, residual malaria transmission is still occurring in some hotspots. To better tailor targeted control actions, it is critical to unravel the underlying environmental and geographical factors that cause the persistence infection in hotspot villages. “Hotspots villages” were defined in our study as those reporting more than six indigenous malaria cases during the previous year. A total of ten villages, including seven hotspots and three non-hotspots, were surveyed. All potential mosquito breeding sites identified in and around the ten study villages were regularly monitored between 2013 and 2017. Monitoring comprised the detection of anopheline larvae and the collection of epidemiological, hydrogeological, topographical, and biogeographical data. The number of larval breeding sites described and monitored during the study period ranged from 50 to 62. Breeding sites were more numerous in hotspot sites in each year of monitoring, with 90.3% (56/62) in 2013, 90.9% (50/55) in 2014, 90.3% (56/62) in 2015 and 86% (43/50) in 2017 (Fisher exact test; p = 1). In the non-hotspot areas, the data for the same years were, respectively, 9.7% (6/62), 9.1% (5/55), 9.7% (6/62) and 14% (7/50) (p = 1). The Hotspot villages were characterized mostly by saline or moderately saline hydro-morphic and halomorphic soils allowing water retention and a potential larval breeding sites. By contrast, non-hotspot villages were characterized mainly by a high proportion of extremely permeable sandy-textured soils, which due to their porosity had low water retention. The annual number of confirmed malaria cases was correlated with the frequency and extent of breeding sites. Malaria cases were significantly more frequent in the hamlets located near breeding sites of *An*. *gambiae* s.l., gradually decreasing with increasing remoteness. This study shows that the characteristics of larval breeding sites, as measured by their longevity, stability, proximity to human habitation, and their positivity in *Anopheles* larvae are likely determining factors in the persistence of malaria hotspots in central-western Senegal. The results of this study shed more light on the environmental factors underlying the residual transmission and should make it possible to better target vector control interventions for malaria elimination in west-central Senegal.

## Introduction

The stubborn persistence of malaria particularly in Sub-Saharan Africa is a global challenge to improved health and sustainable development. The World Health Organization (WHO) estimates that 228 million cases of malaria occurred worldwide in 2018, with nearly 405,000 deaths, of which the main victims were pregnant women and children under 5 years. Noteworthy, 93% of the cases and 94% of the deaths occurring in Africa, and the remainder mainly in the Southeast Asia and the Eastern Mediterranean regions [1].

In Senegal, malaria is endemic with seasonal recrudescence during the rainy season. In 2017, there were 395,706 confirmed malaria cases nation-wide, and 284 deaths [2]. Despite this burden, malaria incidence has declined since 2006, attributable in part to the increase of investments supporting both the prevention and the management of malaria. In the health districts of Mbour, Fatick and Bambey, in the Central Western Senegal, Seasonal Malaria Chemo-prevention (SMC) was implemented between 2008 and 2011, followed by two targeted Indoor Residual Spraying (IRS) campaigns in 2013 and 2014 [3]. These measures were deployed in addition to the standard national strategies rolled out by the National Malaria Control Program (NMCP). Following these interventions, malaria is declining very significantly in these districts, creating a new epidemiological profile in which a few villages experience residual malaria transmission (hotspots villages) next to villages without any detectable transmission [4].

This residual transmission may be due to environmental factors leading to the persistence of larval habitats. In some African countries, larval source management, a strategy which includes larviciding and breeding sites reduction, has been shown to be effective in reducing malaria transmission [5–7]. Information on the spatial distribution of mosquito breeding habitats is needed to enable monitoring and targeting of hotspot areas where vector populations are persistent. The development of Geographic Information Systems (GIS) mapping technology provides high-resolution maps of mosquito distribution or transmission risks, which could improve malaria surveillance system [8,9].

Our study was conducted to investigate the spatial variation and the factors maintaining malaria transmission in some area versus others. More specifically, the study aimed to examine the spatial distribution of vector breeding sites in areas with residual malaria transmission and to measure the correlation between the frequency and distribution of larval habitats and the persistence of residual malaria transmission. Our findings will contribute to better understanding of the geography, biology and ecology of *Anopheles gambiae* s.l. breeding sites, and thus to the application of effective and efficient larval control measures in the central-western Senegal.

## Materials and methods

### Study area

**Fig 1:**
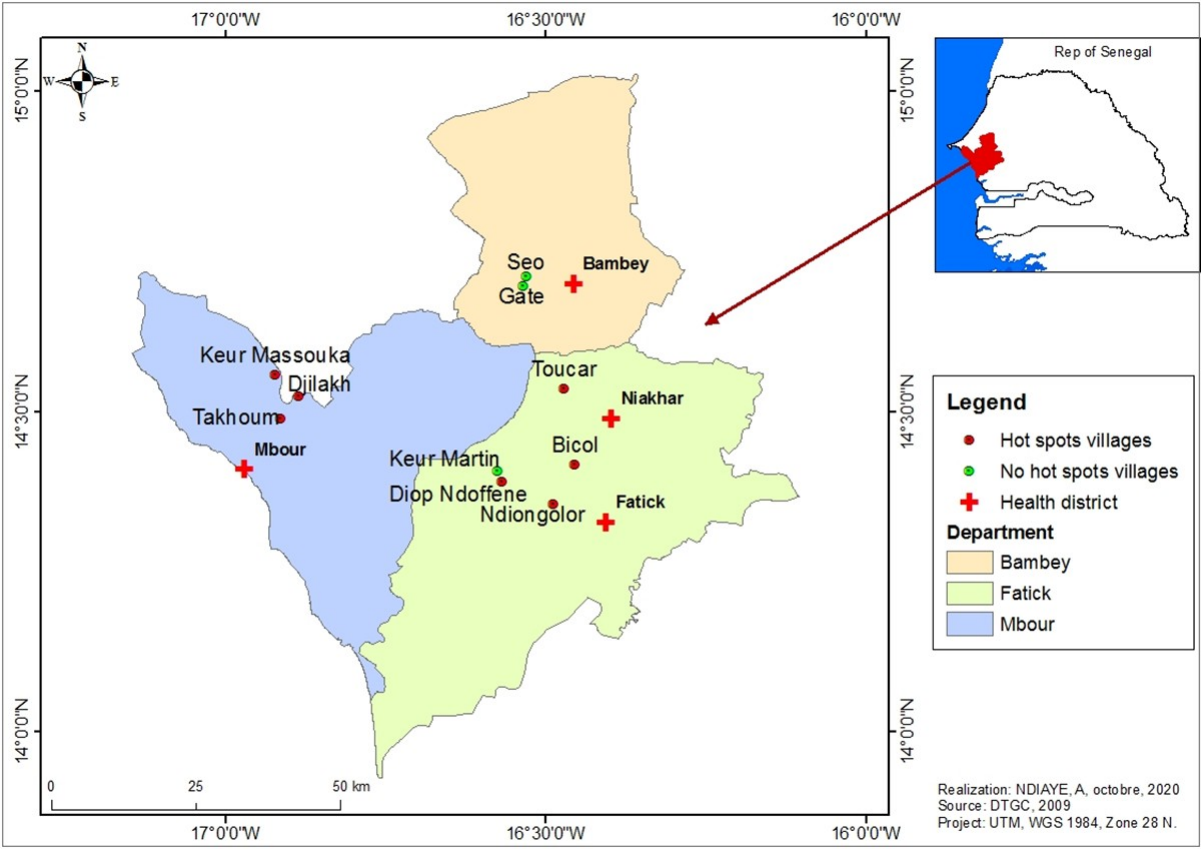
Study area (localization of health districts and epidemiological status of study villages).

The study was carried out in ten villages in central western Senegal (Fig 1). The study area is located between the 13° and 15° 30 ' Northern latitudes and the 13° and 16° 30' Eastern longitudes. It is characterized by a flat, slightly uneven relief, with altitudes rarely exceeding 20 metres [10]. These low highlands are interspersed with valleys in which low lying land, ponds and creeks dry up very quickly during the dry season. As everywhere else in Senegal, the climate of the study area comprises a long dry season between October to May (7 to 8 months) and a very short rainy season between June to September (3 to 4 months). The major climatic features of the north Sudanese biogeographical region result from a combination of geographical and areological factors, with an annual rainfall averages ranging from 400 to 600 mm, unevenly distributed [11]. Monthly average temperatures are high (above 40° C), between April to June. The soil types vary with terrain, bedrock, climate, and hydrography, with different agricultural capabilities, evolving under the influence of human actions. Four types of soil with differences in texture, structure and organic matter content (sandy, clay, clay-sandy and salty soils) were identified in the study area. Located in the Sudan-Sahelian area, the vegetation is very diversified, the geomorphological and pedological characteristics combined with other natural and human related activities explain the density and diversity of the vegetal structure. Indeed, the vegetation is composed of treed, shrubby and herbaceous strata. The dominant economic activity in the area is agriculture, which mobilizes a large workforce, followed by cattle breeding, trade and crafts.

### Selection of study villages

Ten villages were selected on the basis of their epidemiological status as hotspot (7 villages) or non-hotspot (3 villages) (Table 1). Hotspot villages were defined as those reporting more than 6 autochthonous malaria cases during the transmission period (from June to December) the previous year and reported by the health and demographic monitoring system implemented in the area since 2008 by the Department of Medical Parasitology of the Faculty of Medicine, Pharmacy and Stomatology of the University Cheikh Anta Diop of Dakar [4].

**Table 1:** General characteristics of hotspot versus non-hotspot areas selected.

Village	Epidemilogical statut	Types of soil mainly encountered	Types of crops developed in the area	Presence of animals	Nature and characteristics of dominant Anopheles breeding sites	Average distance between breeding sites and houses	Species of anopheles present in the area	Control measures
Djilakh	Hotspot	Sandy, clayey and clayey-sandy soils	Millet, sorghum, maize, peanuts and market gardening	Yes	Naturel/ temporary	365 m	Ag; Af; Ap; Ar	IRS-LLINs-SMC
Takhoum Ndoundour	Hotspot	Sandy, clayey and clayey-sandy soils	Millet, sorghum, maize, peanuts and market gardening	Yes	Naturel/ temporary	500 m	Ag; Af; Ap; Ar	IRS-LLINs-SMC
Keur Massouka	Hotspot	Sandy, clayey and clayey-sandy soils	Millet, sorghum, maize, peanuts and market gardening	Yes	Naturel/ temporary	460 m	Ag; Af; Ap; Ar	IRS-LLINs-SMC
Toucar	Hotspot	Clayey and clayey-sandy soils	Millet, sorghum, maize, peanuts and market gardening	Yes	Naturel/ temporary	200 m	Ag; Af; Ap; Ar	IRS-LLINs -SMC
Bicol	Hotspot	Clayey and clayey-sandy soils	Millet, sorghum, maize, rice and peanuts	Yes	Naturel/ temporary	158 m	Ag; Af; Ap; Ar	IRS-LLINs-SMC
Ndiongolor	Hotspot	Sandy, clayey and clayey-sandy soils	Millet, sorghum, maize, rice, peanuts and market gardening	Yes	Naturel/ temporary	105 m	Ag; Af; Ap; Ar	IRS-LLINs-SMC
Diop Ndoffene	Hotspot	Sandy, clayey-sandy and salty halomorphic soils	Millet, sorghum, maize and peanuts	Yes	Naturel/ temporary	197 m	Ag; Af; Ap; Ar	IRS-LLINs-SMC
Keur Martin	Non-hotspot	Sandy, clayey-sandy and salty halomorphic soils	Millet, sorghum, maize and peanuts	Yes	Naturel/semi-permanent	170 m	Ag; Af; Ap; Ar	LLINs
Gate	Non-hotspot	Sandy and clayey-sandy soils	Millet, sorghum, maize and peanuts	Yes	artificial / temporary	100 m	Ag; Af; Ap; Ar	LLINs
Séo	Non-hotspot	Sandy and clayey-sandy soils	Millet, sorghum, maize and peanuts	Yes	artificial / temporary	350 m	Ag; Af; Ap; Ar	LLINs

Ag: *Anopheles gambiae* s.l.; Af: *Anopheles funestus*; Ap: *Anopheles pharonensis*; Ar: *Anopheles rufipes*.

LLINs: Long-lasting insecticidal nets.

IRS: Indoor Residual Spraying.

SMC: Seasonal Malaria Chemoprevention.

### Data collection

Data collected comprised the geographical location and environmental factors prevailing in each of the selected study villages, as well as physical, chemical and biological parameters of encountered larval breeding. Larval breeding sites were monitored monthly in the rainy season, from July to December, and bimonthly in the dry season, between January and June. The study was approved by the National Ethics Committee of Senegal and no other specific permissions were required.

### Characterization and mapping of larval breeding

The localization and the survey of the larval breeding sites within and around hotspot and non-hotspot villages were characterized annually during the raining season from 2013 to 2017. In each of the selected villages, all the surface-water bodies likely to harbor *Anopheles gambiae* s.l. larvae were visually checked to assess the presence of larvae. When the presence of larvae was confirmed, we recorded the following for each positive breeding site: hydrogeological, biogeographical and topographical information, including their coordinates using Geographic Positioning System (GPS) machine, Garmin International, GPSMAP® 60CSx [12,13]. As consequence, all the encountered surface water bodies within and around the hotspot and non-hotspot villages which could potentially harbor anopheline larvae were identified, mapped, then followed up over the time to assess their spatial distribution, their temporal functioning and their role in the maintenance of residual malaria transmission.

We constructed a thematic map for the hotspot village of Djilakh, using the thematic layers of the flora, the type of soil, the location of the water bodies (GPS coordinates), their distance to the nearest human dwellings, the type and putative origin of the water filling each water body. Geographical data on malaria cases and those collected on larval breeding sites were merged to form a database called Geographical Information System (GIS). The mapping software QGIS Desktop 2.2.0 was used for the elaboration of maps and spatial analyses.

### Mosquito larvae collection

Mosquito larvae were collected from each positive water body found within and nearby each of the study villages using the "dipping" method [14]. The larvae were identified as *Anophelinae versus Culicinae* (*Culex* and *Aedes*) based on their resting position in water and their stage of larval development (L1-L2-L3 and L4) [15]. Anopheline larvae and nymphs collected from breeding sites were reared until emergence. They were then further identified at the species level using morphological identification keys [16]. Because the study’s main objective was to map malaria vectors’ breeding habitats, we discarded *culicinae* larvae. The anopheline larval density within each positive breeding site was calculated by counting the number of larvae per liter of water.

### Collection of morbidity data

Malaria morbidity was estimated from confirmed malaria cases using rapid diagnostic tests (RDTs) results for each of the ten villages for a period from the 1^st^ January 2013 to the 31^st^ December 2017. Data, including the age, the sex, RDT tests results (as positive or negative), the code and origin of patient, were retrieved from the consultation registers archived at the health post of each study village. All sensitive information about patients was anonymized to comply the personal data protection regulations. Finally, morbidity data were collated with the geographical coordinates of all the districts and health post visited for mapping purpose. It should also be noted that not all malaria cases are registered at the health post because some patients stay at home, which is a limitation to the collection of malaria morbidity data. In the health posts of Bicol and Diop Ndoffene, the consultation registers for the years 2013, 2014 and 2015 were not found.

### Climatic data collection

Rainfall is a key factor in determining mosquito larval populations. We accessed rainfall data at the health district level from the records of the National Agency of Civil and Meteorological Aviation (ANACIM). We selected rainfall data from the rainfall stations located in the district of Mbour, Fatick and Bambey, corresponding to our study areas.

### Data processing and analysis

The geographic data of all GPS-tagged breeding sites and other information collected from the field were quality-checked before analysis. Only data that met good quality criteria, in terms of reliability of the source and data completeness, were used for mapping and further analysis in Excel 2013, Epi info 7 and QGIS software. Data on larval breeding sites and human settlements were imported into the QGIS Desktop 2.2.0 mapping software to drawn maps and for spatial analysis.

## Results

### Relationship between the soil type and the frequency of breeding sites in hotspot and non-hotspot villages

The number of larval breeding sites inventoried and monitored between 2013 and 2017 ranged from 50 to 62 throughout the study area. In the hotspot villages, the natural breeding sites were much more numerous than the artificial ones, with 40 and 11 respectively. Natural breeding sites correspond to puddles and temporary pools of water. Artificial breeding sites, caused by open excavations of sand quarries, were found in all hotspots areas except two villages (Diop Ndoffene and Takhoum Ndoundour). At the non-hotspots areas, we have two semi-permanent breeding sites located in the village of Keur Martin and 4 other artificial ones.

The number of larval breeding sites was higher, with no significant difference over the years in hotspots areas with 90.3% (56/62) in 2013, 90.9% (50/55) in 2014, 90.3% (56/62) in 2015 and 86% (43/50) in 2017 (Fisher exact test; p = 1). In the non-hotspot areas, the data for the same years were, respectively, 9.7% (6/62), 9.1% (5/55), 9.7% (6/62) and 14% (7/50) (p = 1).

Across the study area, both the occurrence and the abundance of anopheline larval breeding sites were most strongly correlated with rainfall and soil characteristics. While in the non-hot spot villages the soils were mainly halomorphic with more or less a clay texture, those in the hotspot areas were of tropical ferruginous types with little leaching with fine texture and high proportion of silt, and a fairly high content of clay, hence increasing their capacity for water retention. Moreover, in term of the level of the deposits at the immediate surroundings areas, hydromorphic soils with a higher rate of clay than the halomorphic ones were most frequent. These types of soils were found mainly in the villages of Bicol, Ndiongolor and Toucar (Fig 1). They were also present in the north of the village of Keur Masssouka and throughout the eastern parts of Djilakh and Takhoum Ndoundour. On the other hand, the halomorphic soils were characterized by the presence of a denatured surface and were more often connected to the ramifications of the inlets. These types of soil were found in Diop Ndoffene and the non-hotspot village of Keur Martin.

In the non-hotspot villages only few larval breeding sites (10.48%) were found (Table 2), consisting mainly by borrow pits. Moreover, in Gate, the anthropogenic breeding sites consisted of water-filled tyre tracks left by heavy vehicles mired during the construction of the lateritic track connecting the village to the national road. These type of water bodies, are characterized by a high proportion of sand-textured soils that are extremely porous, with low water retention. Their texture is unstable and they dry up very quickly after the rains stop. These types of soils are very common in Gate and Seo, where no natural anopheline breeding sites were found. Noteworthy, only two natural breeding sites, named Ndiangothie and Sakh maak, were found in the non-hotspot villages. They were located in Keur Martin (Fig 1).

**Table 2:** Number of breeding sites surveyed by village from 2013 to 2017.

			Number of *An*. *gambiae* s.l. larval breeding sites per year			
Health districts	Villages	Epidemiological statut	2013	2014	2015	2017
Mbour	Djilakh	Hotspot	9	9	9	5
	Takhoum Ndoundour		2	2	2	2
	Keur Massouka		5	5	5	3
Fatick	Keur Martin	Non-hotspot	2	2	2	4
	Diop Ndoffene	Hotspot	7	7	7	7
	Bicol		9	9	9	7
	Ndiongolor		15	10	15	12
Niakhar	Toucar	Hotspot	9	8	9	7
Bambey	Gate Diokoul 2	Non-hotspot	2	1	2	1
	Seo		2	2	2	2
Total in hotspot area			56	50	56	43
Total in non-hotspot area			6	5	6	7

### Correlation between rainfall and vector breeding sites frequency

**Fig 2:**
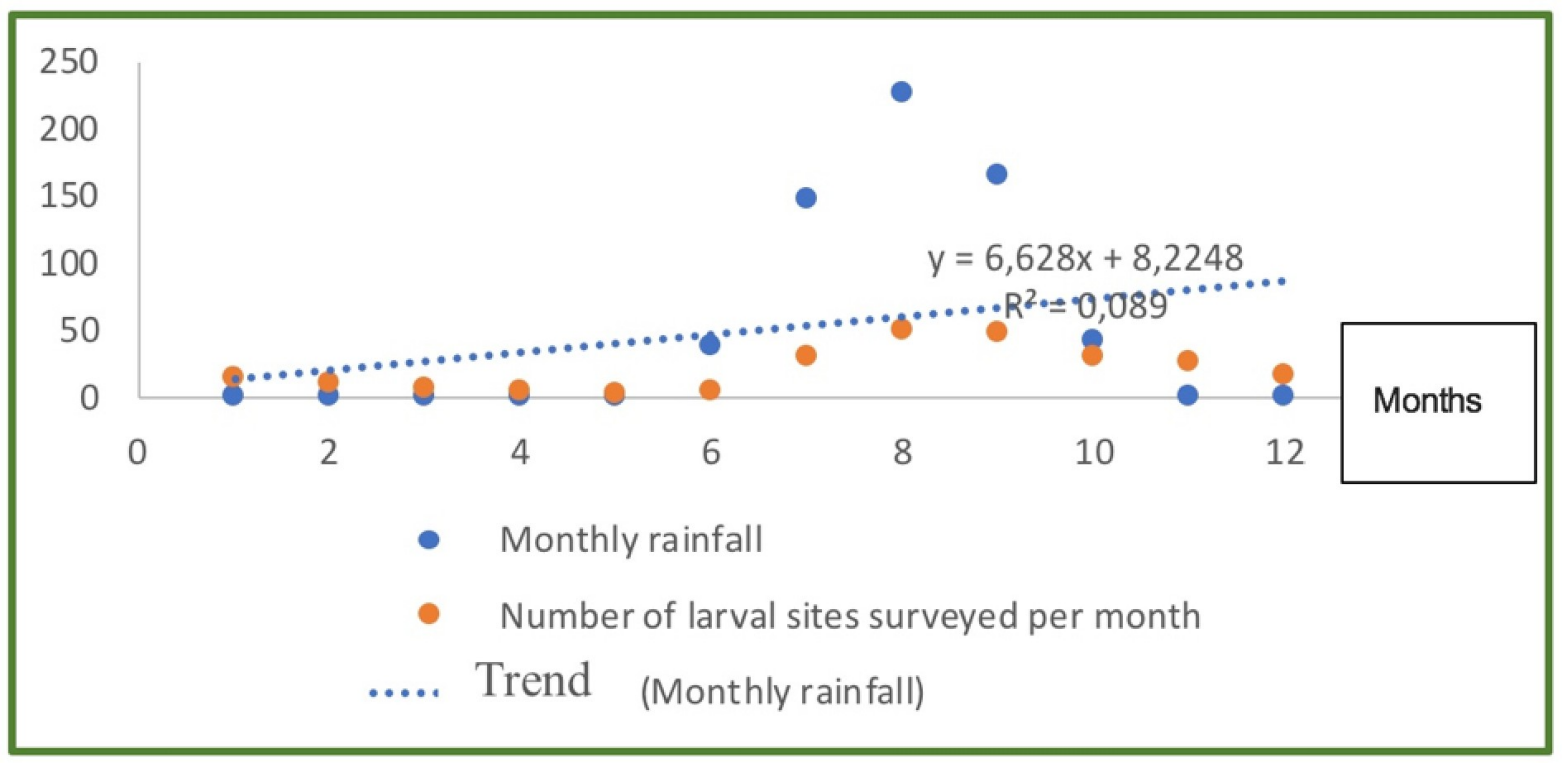
Evolution of the monthly average index of the frequency of *Anopheles gambiae* s.l. breeding sites according to monthly rainfall.

In the study villages, the occurrence of breeding sites depended on the frequency and amount of rain. From 2013 to 2017, a total of 62 breeding sites were surveyed across all the study area (Table 2). The number and size of known and surveyed breeding sites varied between the beginning and the end of the rainy season. Overall there was a positive correlation (r = +0.089) between the rainfall and the proliferation of the breeding sites (Fig 2). In July, when the first rains were recorded in the area, only 67.7% (42/62) of the surveyed surface-water body sites were filled with water, varying in size between 1.5 to 17,000 m^2^. In the middle of the rainy season, corresponding to the peak rainfall between August and September, the number and size of the surface-water bodies were the highest, with 96% of the known breeding sites full, and their surfaces varying between 25 and more than 200,000 m^2^. By the end of the rainy season in October, the percentage of the breeding sites dropped to 61% as well as their size varying between 3 and 25,000 m^2^. Unsurprisingly, the number and size of surface water bodies increased not only with rainfall, but also according to the characteristics of the substrate. The frequency of breeding sites larger than 20,000 m^2^ were much higher in non-hotspot villages than in hotspot villages. Thus, taking the average number of the breeding sites surveyed during this study, the frequency of larger ones (>20,000 m^2^) was 83.3% (5/6) in the non-hotspots areas and 49% (25/51) in the hotspots areas. Breeding sites smaller than 500 m^2^ represented 45% (23/51) in the hotspots and 16.6% (1/6) in non-hotspot villages. In hotspot areas breeding sites of size between 500 m^2^ and 20,000 m^2^ were not found, whereas in the non-hotspot areas larval sites of this size represented 5.8% (3/51).

### Relationship between morbidity, frequency and size of breeding sites in the different epidemiological settings

Across the ten study villages, the number and size of *An*. *gambiae* larval breeding sites have varied over the study years. Overall the number of the breeding sites was 62 in 2013, 55 in 2014, 62 in 2015 and 50 in 2017 (Table 2). In the meantime, the overall malaria incidence varied with 0.109‰ in 2013, 0.064‰ in 2014, 0.142‰ in 2015 and 0.036‰ in 2017.

In general, the annual malaria morbidity rate was consistent with the frequency of larval breeding sites. Morbidity was higher in 2013 (26.1%) and in 2015 (35.2%), consistent with the significant rainfall recorded in the area for both years. Analysis of the frequency of confirmed malaria cases shows a similar evolution to that of the number of breeding sites surveyed. We also observed that the highest number of malaria cases was recorded in the hotspot villages where the maximum number of breeding sites (50) were found. By contrast, there were fewer cases in the non-hotspot villages with 7%, where breeding sites were also less frequent (Table 3). The small percentages of malaria cases in the hotspot villages of Bicol with 4.7% and Diop Ndoffene with 2.1% could be explained by the absence of morbidity data for the years 2013, 2014 and 2015, due to the loss of some consultation registers.

**Table 3:** Number of confirmed malaria cases by village from 2013 to 2017.

			Number of annual malaria cases				
Health districts	Villages	Epidemiological statut	2013	2014	2015	2016	2017
Mbour	Djilakh	Hotspot	35	14	53	19	4
	Takhoum Ndoundour		12	12	21	4	5
	Keur Massouka		20	5	11	3	1
Fatick	Keur Martin	Non hotspot	0	0	12	2	1
	Diop Ndoffene*	Hotspot	NA	NA	NA+5	3	1
	Bicol*		NA	NA	NA	11	9
	Ndiongolor		22	20	25	12	5
Niakhar	Toucar	Hotspot	14	12	16	5	9
Bambey	Gate Diokoul 2	Non hotspot	5	1	4	2	1
	Seo		1	0	0	0	0
Total in hotspot area			103	63	131	57	34
Total in non-hotspot area			6	1	16	4	2

* Villages with missing data.

NA: Not Available.

In two villages, Keur Martin and Diop Ndoffene, breeding sites typically were large natural depressions of sizes up to over 200000 m^2^, most often temporary, filled by rainfall and overflowing seawater. These runoff inputs from marine or fluvial waters affected the salinity of the neighbouring breeding sites, with repercussions for the presence, density and composition of anopheline larvae and, as a consequence, for malaria morbidity (Table 4).

**Table 4:** Positivity in *An*. *gambiae* s.l. larvae of breeding sites and presence of malaria cases in different epidemiological settings.

Epidemilogical statut	Year	Village	Number of breeding sites	Positive in *An*. *gambiae* s.l. larvae	Negative in *An*. *gambiae* s.l. larvae	Positivity (%)	Number of annual malaria cases
Hotspot	2013	Djilakh	9	7	2	77,7%	35
Hotspot		Takhoum Ndoundour	2	1	1	50%	12
Hotspot		Keur Massouka	5	2	3	40%	20
Hotspot		Toucar	9	9	0	100%	14
Hotspot		Bicol	9	8	1	88,8%	NA
Hotspot		Ndiongolor	15	13	2	86,6%	22
Hotspot		Diop Ndoffene	7	4	3	57,1%	NA
Non hotspot		Keur Martin	2	2	0	100%	0
Non hotspot		Gate Diokoul 2	2	2	0	100%	5
Non hotspot		Seo	2	1	1	50%	1
**Total in hotspot area**			**56**	**44**	**12**	**78,5%**	**103**
**Total in non-hotspot area**			**6**	**5**	**1**	**83,3%**	**6**
Hotspot	2014	Djilakh	9	5	4	55,5%	14
Hotspot		Takhoum Ndoundour	2	1	1	50%	12
Hotspot		Keur Massouka	5	2	3	40%	5
Hotspot		Toucar	8	7	1	87,5%	12
Hotspot		Bicol	9	6	3	66,6%	NA
Hotspot		Ndiongolor	10	8	2	80%	20
Hotspot		Diop Ndoffene	7	3	4	42,8%	NA
Non hotspot		Keur Martin	2	1	1	50%	0
Non hotspot		Gate Diokoul 2	1	1	0	100%	1
Non hotspot		Seo	2	2	0	100%	0
**Total in hotspot area**			**50**	**32**	**18**	**64%**	**63**
**Total in non-hotspot area**			**5**	**4**	**1**	**80%**	**1**
Hotspot	2015	Djilakh	9	8	1	88,8%	53
Hotspot		Takhoum Ndoundour	2	2	0	100%	21
Hotspot		Keur Massouka	5	4	1	80%	11
Hotspot		Toucar	9	9	0	100%	16
Hotspot		Bicol	9	8	1	88,8%	NA
Hotspot		Ndiongolor	15	14	1	93,3%	25
Hotspot		Diop Ndoffene	7	5	2	71,4%	NA+5
Non hotspot		Keur Martin	2	2	0	100%	12
Non hotspot		Gate Diokoul 2	2	2	0	100%	4
Non hotspot		Seo	2	1	1	50%	0
**Total in hotspot area**			**56**	**50**	**6**	**89,2%**	**NA+131**
**Total in non-hotspot area**			**6**	**5**	**1**	**83,3%**	**16**
Hotspot	2016	Djilakh	NA	NA	NA	NA	19
Hotspot		Takhoum Ndoundour	NA	NA	NA	NA	4
Hotspot		Keur Massouka	NA	NA	NA	NA	3
Hotspot		Toucar	NA	NA	NA	NA	5
Hotspot		Bicol	NA	NA	NA	NA	11
Hotspot		Ndiongolor	NA	NA	NA	NA	12
Hotspot		Diop Ndoffene	NA	NA	NA	NA	3
Non hotspot		Keur Martin	NA	NA	NA	NA	2
Non hotspot		Gate Diokoul	NA	NA	NA	NA	2
Non hotspot		Seo	NA	NA	NA	NA	0
**Total in hotspot area**			NA	NA	NA	NA	**57**
**Total in non-hotspot area**			NA	NA	NA	NA	**4**
Hotspot	2017	Djilakh	5	5	0	100%	4
Hotspot		Takhoum Ndoundour	2	2	0	100%	5
Hotspot		Keur Massouka	3	2	1	66,6%	1
Hotspot		Toucar	7	7	0	100%	9
Hotspot		Bicol	7	7	0	100%	5
Hotspot		Ndiongolor	12	11	1	91,6%	9
Hotspot		Diop Ndoffene	7	4	3	57,1%	1
Non hotspot		Keur Martin	4	3	1	75%	1
Non hotspot		Gate Diokoul 2	1	1	0	100%	1
Non hotspot		Seo	2	1	1	50%	0
**Total in hotspot area**			**43**	**38**	**5**	**88,3%**	**34**
**Total in non-hotspot area**			**7**	**5**	**2**	**71,4%**	**2**

NA: Not Available.

### Evolution of malaria cases according to larval breeding sites frequency in hotspots and non-hotspots villages

**Fig 3:**
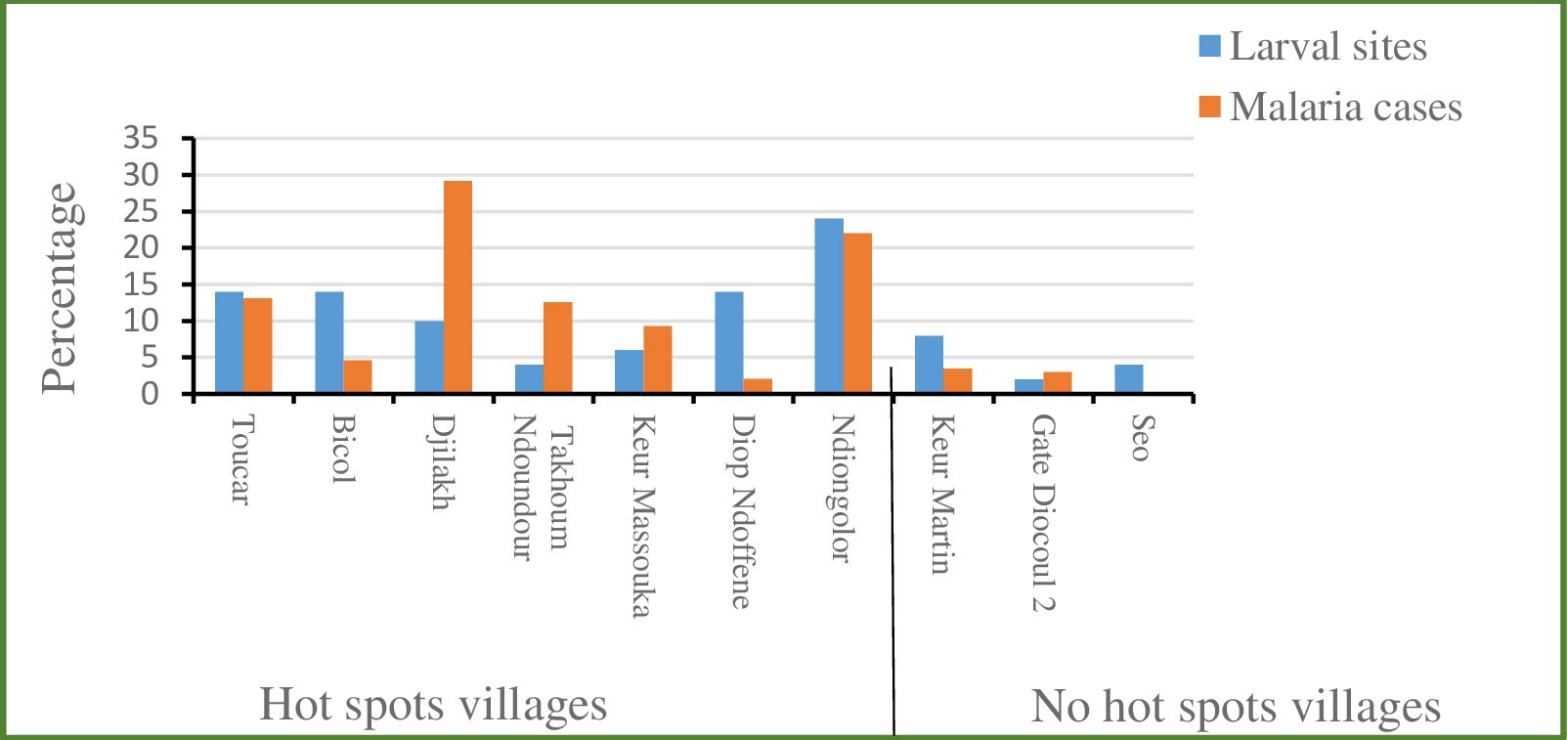
Relationship between morbidity and frequencies of *An*. *gambiae* sl breeding sites in hotspot and non-hotspot villages between 2013 and 2017.

**Fig 4:**
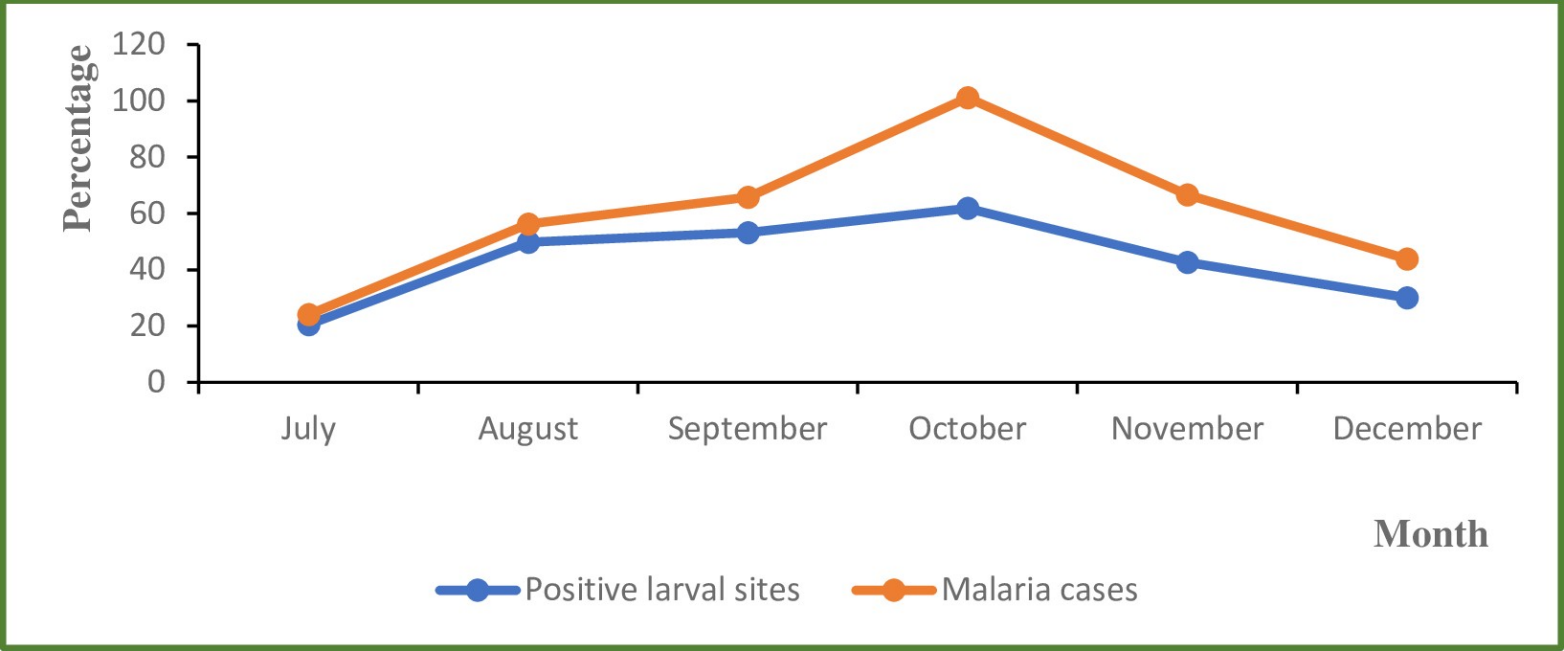
Relationship between monthly evolution of malaria cases and positivity of *An*. *gambiae* s.l. larval breeding sites between 2013 to 2017 in the ten study villages.

All *Anopheles* larvae collected were morphologically identified as *Anopheles gamb*iae s.l. The frequency of *An*. *gambiae* s.l. breeding sites varied over the rainy season, with an impact on malaria incidence both in hotspots and non-hotspots villages (Fig 3). The highest density of larvae was recorded in October and was most marked in hotspots villages, with an average rate of 2 larvae per liter sampled water. Density was twenty-five time lower in non-hotspots villages, with an average rate of 0.08 larvae per liter. Overall malaria morbidity and vector breeding were positively correlated (r^2^ = 0.63). Moreover, the two parameters displayed similar trends over the time in the hotspot villages as well as in the non-hotspot villages. Overall at the beginning of the rainy season in July, the average *Anopheles gambiae* s.l. breeding sites found was 20.5%, rising to 53.2% in the middle of the rainy season in September, and 61.8% at the end of the rainy season in October (Fig 4). More than the half (51.87%) of the malaria cases recorded in the whole study area occurred between September and October, coinciding with the peak of larval positivity at the breeding sites.

### Variation of malaria morbidity according to the proximity of breeding sites and human dwellings: The case of the hotspot village of Djilakh

**Fig 5:**
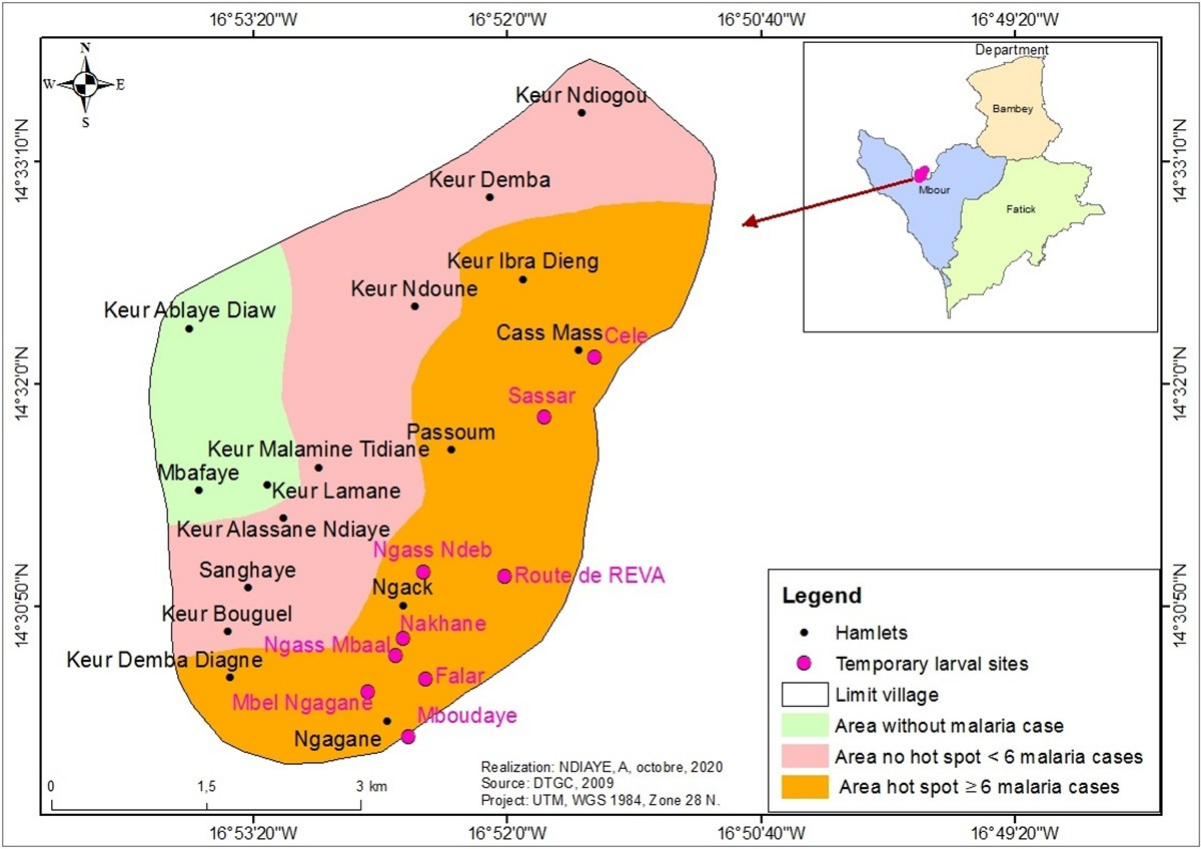
Malaria risk in Djilakh area: High risk of morbidity (brown), medium risk (pink) and risk-free (green).

In the village of Djilakh in the health district of Mbour, which comprises a group of hamlets, analysis of morbidity data according to the location of *An*. *gambiae* s.l. larval breeding sites in relation to dwellings revealed three geographical areas displaying different level of the disease eco-epidemiology (Fig 5).

Overall, malaria cases were significantly higher in the hamlets located in the eastern part of the village closest to breeding sites (< 500m) with 82.4% (103/125), followed by the hamlets located in the central western part of the village between 500 to 1000 meters, with 17.6% (22/125), and finally those in the western part farer away (≥1000m), where no cases were recorded during the study period (P < 0.05).

The soil composition was different across the village, being mainly constituted by hydromorphic soils with a fairly high contain of clay, hence increasing its water retention capacity. Looking at the soils’ composition, only the eastern part of the village consists of hydromorphic clay soils, while the rest of the study site consist of tropical ferruginous soils with little or no leaching (known locally as "dior" soils). These soils have a sandy texture and are very permeable, with low water retention capacity.

## Discussion

Diverse ecological and environmental factors determine malaria transmission and epidemiology across the study area. The primordial biotope for the proliferation of mosquitoes results from the interaction between several environmental components such as water, the structure and composition of the soils and the vegetation associated with them [17,18]. Human activity also plays a key role in influencing malaria transmission and geographical distribution. Human communities differ in how and the extent to which they create suitable or unfavorable conditions for malaria to spread and persist [19].

The mineralogical composition of soils can affect the proliferation of putative mosquito breeding sites, especially when combined with significant rainfall [20]. The creation of anophelines larval breeding sites is largely related to the nature of the soil substrates. This can explain why more breeding sites were found in the hotspot villages of Ndiongolor, Toucar, Bicol and Diop Ndoffene, where the soils consist mainly of hydromorphic and the saline halomorphic types. Soil type can also explain the observed residual transmission in these villages. More specifically, hydromorphic and the halomorphic soils, beside their capacity to retain water over a long period of time, are also suitable for larval development of *Anopheles* species such as *An*. *arabiensis* and *An*. *melas* according to their salinity level. Indeed, in the localities where we found hydromorphic soils with lasting longer surface water, conditions were favorable for *An*. *gambiae* s.l. larvae, making these localities at high risk of malaria [21]. Conversely, in the non-hotspot villages of Gate, Diocoul and Seo, the leached tropical ferruginous type of soils did not allow surface water bodies to last for long due to the significant infiltration of rain water. Consequently, fewer and very fast drying surface water points were found in those study sites. This finding can explain why there were relatively few breeding sites recorded inside and around these villages and, consequently, the lower levels of malaria incidence.

The existence of suitable breeding sites lasting long enough for complete larval development is fundamental to the life cycle of malaria vectors, and consequently to the transmission of the disease. Larval development is directly influenced by a large number of variables. These include the volume of the water at breeding sites and the dynamics, and physical and chemical characteristics of the water, its solar exposure, the presence/absence and the type of vegetation, occupation of the territory, and also by the average rainfall, water infiltration, evapotranspiration, and the water retention capacity of the soil [22–24].

Most of the putative anopheline breeding sites are water-filled during the raining season, corresponding to the malaria transmission season, when vector populations proliferate. Across the study area, malaria transmission lasts for 5 to 6 months (from July to December) and is maintained by three main vectors species belonging to the Gambiae complex: *An*. *arabiensis*, *An*. *coluzzii* and *An*. *melas* [25–27]. However, other competent species such as *An*. *funestus*, *An*. *gambiae* may be involved in transmission where the larval ecology is favorable. As usually observed elsewhere in Senegal, malaria incidence increased progressively immediately after the first rains with a peak at the middle of the rainy season toward the month of October, while the peak of the rain was recorded earlier between August and September. This led to proliferation of surface water collections suitable for the anopheline larval development [28]. This is a well-described situation in which when rains increase and are recurrent, as observed in the months of August and September, breeding sites become constantly flooded, and the leaching of the breeding sites cause the death of immature larval stages [29]. Then, when rainfall episodes become more spaced, from September toward October, breeding sites become more stable and productive, consequently changing disease transmission dynamics. Indeed, our study has shown that the peak of morbidity follows with two months delay the peak of rains. The study also confirms previous observations in the area reporting an equivalent lag time of two months [30]. Indeed, the larval development and abundance of malaria vector are closely related to the presence and productivity of breeding sites, which for the members of the *An*. *gambiae* complex are partly dependent on rainfall dynamics [31].

Having clearly demonstrated the link between malaria morbidity and the number of productive breeding sites in the study villages, the rainfall deficit observed in October 2014 can explain the scarcity of breeding sites and lower malaria-vector density across the study area. Indeed, the large majority of known breeding sites dried up very quickly due the decrease of rains toward the end of September 2014 [25]. This resulted in a drop in malaria morbidity observed that year, with 45 fewer confirmed malaria cases than in the previous year (2013).

Among factors highly influencing the abundance of anopheline mosquitoes, are the following: the thermal and hygrometric, physical and chemical parameters of the breeding sites water, are of the highest importance. Each mosquito species has its specific set of requirements for water properties for optimal larval development. Although the physical and chemical parameters of the water vary over the time and with the rainfall dynamic, they become more stable towards the end of the rainy season with breeding sites less diluted by recurrent rainfalls, thus favoring of mosquito proliferation [32]. Indeed, in the Sudanese zone of West Africa, the peak of transmission is usually recorded towards the end of the rainy season when the humidity remains high and the rains are less frequent, no longer disturbing breeding site parameters, including their physical and chemical factors [33,34].

Our analysis of the breeding site location to human dwellings showed that malaria cases decrease with distance from observed breeding sites [35,36]. For instance, in the village of Djilakh, the number of new malaria cases was much higher in the hamlets nearby breeding sites, where the soil type was more favorable to rainwater retention. The number of cases decreased gradually with distance. Consequently, the areas most exposed to the risk of malaria transmission can be considered as those close to the main water retention areas and the most remote neighborhoods with soils that retain less water are the least exposed [21,37].

## Conclusion

The results of this study showed that the dynamics of anopheles mosquitoes breeding sites with regard to their abundance, stability and proximity to houses strongly influence the epidemiology and the persistence of malaria transmission in hotspots villages in central western Senegal. We conclude that a vector management strategy, targeted to larval breeding sites in hotspot villages, aiming at reducing the number of mosquito immature stages, could be integrated as an effective additional measure in this area where almost all functioning breeding sites are known. The outcome could be to further reduce transmission and achieve the goal of malaria elimination in central western Senegal.

## Supporting information

S1 File (ZIP)

S2 File (RAR)
