# Supplementary figures and images for: Mapping the breeding sites of Anopheles gambiae s. l. in areas of residual malaria transmission in central western Senegal
Source: PLoS One. 2020 Dec 11;15(12):e0236607. doi: 10.1371/journal.pone.0236607 (PMC7732347; doi:10.1371/journal.pone.0236607)

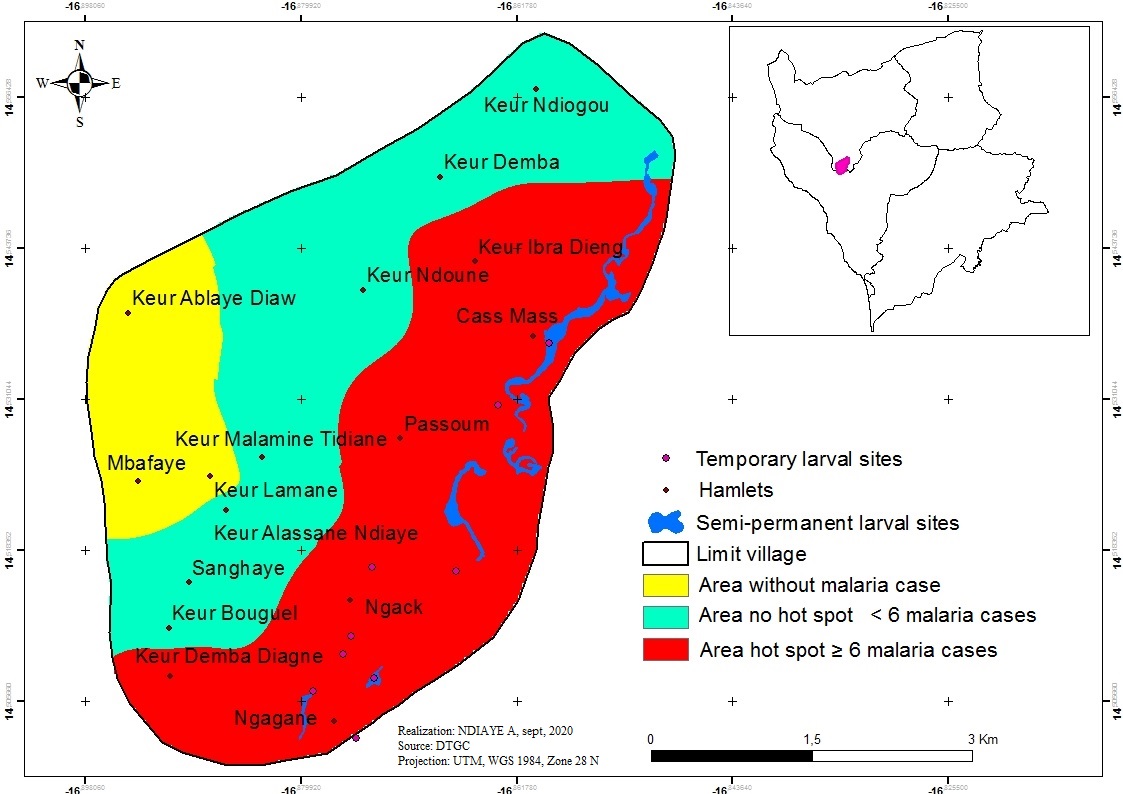

Supplement: S1 File — (ZIP) [file pone.0236607.s001.zip › PROJET VILLAGE DE DJILAKH/Djilakh.jpg]
